# The association of diabetic retinopathy and diabetic kidney disease in patients with type 2 diabetes mellitus: a prospective observational research study

**DOI:** 10.3389/fendo.2025.1473517

**Published:** 2025-09-01

**Authors:** Yutong Wu, Zhuomin Hong, Xiangxia Luo

**Affiliations:** Gansu Provincial Hospital of Traditional Chinese Medicine (TCM), Gansu University of Chinese Medicine, Lanzhou, Gansu, China

**Keywords:** type 2 diabetes mellitus, diabetic retinopathy, diabetic kidney disease, traditional Chinese medicine syndrome type, clinical relationship

## Abstract

**Background:**

Diabetic retinopathy (DR) and diabetic kidney disease (DKD) are two prevalent diabetic complications significantly influencing global health and quality of life. However, little is known about their relationship in patients with type 2 diabetes mellitus (T2DM).

**Aim:**

This study aimed to investigate the relationship between DR and DKD through a prospective observational research study.

**Patients and methods:**

T2DM patients were recruited from November 2020 to November 2022. A total of 223 T2DM patients were finally enrolled. Additionally, 50 healthy examinees were included as the control group. Diagnostic and staging criteria for DR are based on previously established standards. DKD was assessed using serum levels of cystatin C (CysC), β2-microglobulin (β2-MG), and homocysteine (Hcy). Traditional Chinese medicine (TCM) syndrome differentiation standards were based on the TCM Diagnosis and Treatment Standards for diabetes Retinopathy issued by the diabetes Branch of the Chinese Society of Traditional Chinese Medicine in 2011. The outcomes of interest were measured at baseline.

**Results:**

This study included 223 T2DM patients aged from 32 to 78 years. According to DR staging, patients were further categorized into four T2DM subgroups, namely, T2DM without DR, T2DM with light or moderate non-proliferative DR (lmNPDR), T2DM with heavy non-proliferative DR (hNPDR), and T2DM with proliferative DR (PDR). Among the participants, 154 (154/223, 69.06%) were diagnosed with DR. As DR severity increased, the levels of three DKD indicators presented a significant upward trend compared to those in the control group, except for Hcy in the PDR subgroup. Spearman’s correlation analysis revealed significant associations between each DKD indicator and the T2DM subgroups, with rs = 0.223, p = 0.001, for Hcy; rs = 0.452, p < 0.001, for CysC; and rs = 0.564, p < 0.001, for β2-MG. Three TCM syndrome types were identified in the 223 T2DM patients. For four T2DM subgroups, the proportion of qi–yin deficiency increased with decreasing DR severity, whereas the proportion of yin–yang deficiency increased with increasing DR severity. Three DKD indicators exhibited statistically significant differences between the deficiency of the liver and kidney or yin–yang deficiency and qi–yin deficiency, except for Hcy in yin–yang deficiency.

**Conclusion:**

This study suggests a potential association between DR and DKD and provides evidence that the incidence of qi–yin deficiency and yin–yang deficiency exhibits opposite trends with respect to DR severity.

## Introduction

Diabetes has become a critical global public health issue, significantly affecting human health and quality of life (1). Type 2 diabetes mellitus (T2DM) is the most prevalent subtype of diabetes, accounting for over 85% of all diabetic cases (2). Patients with diabetes are at risk of developing macrovascular complications, such as cardiovascular diseases (CVDs), and microvascular complications, including diabetic retinopathy (DR), diabetic kidney disease (DKD), and diabetic peripheral neuropathy (DPN) (3). As microvascular complications, DR and DKD are of particular clinical concern due to their progressive nature and severe consequences, such as vision loss, end-stage renal disease, and increased mortality in T2DM patients (4, 5). Given their significant burden, plenty of work is urgently needed to better understand and effectively treat DR and DKD in T2DM patients.

DR and DKD not only share similar risk factors related to microcirculatory damage but also exhibit parallel patterns in their onset and progression (5, 6). Despite these similarities, there are certain differences in their clinical manifestations and underlying pathological mechanisms, and their relationships remain incompletely understood in clinical practice (5, 6). Previous studies have found that serum levels of cystatin C (CysC), β2-microglobulin (β2-MG), and homocysteine (Hcy) are significantly more sensitive than serum creatinine (SCr) and blood urea nitrogen (BUN) for detecting early-stage DKD, providing a more accurate reflection of glomerular filtration function and the extent of renal damage in patients (7, 8). However, whether these biomarkers can serve as effective indicators for characterizing the occurrence of DR and evaluating the severity of diabetes-induced eye damage has not yet been reported.

According to the China Association of Traditional Chinese Medicine (TCM) in 2011, TCM syndromes for DR can be categorized into three types, namely, qi and yin deficiency, liver and kidney deficiency, and yin and yang deficiency (9). Previous studies have demonstrated that clinical treatments could effectively alleviate DR based on TCM syndromes of diabetic patients (10, 11). Given the clinical similarities and shared characteristics of DR and DKD, uncovering the association between TCM syndromes and DKD helps to estimate the theory of “the same treatment for different diseases” by treating DR and DKD by combining TCM syndromes.

The aim of this study was to investigate the relationship between DR and DKD and to explore the roles of TCM syndromes in T2DM patients through a prospective observational research study. The findings are expected to offer novel insights into clinical strategies for treating DR and DKD in T2DM patients.

## Materials and methods

### Sample size estimation

The sample size of the study was calculated to ensure adequate statistical power using the PASS software (V15.0, NCSS, LLC, Kaysville, UT, USA). For three independently analyzed indicators (Hcy, β2-MG, and CysC) related to DKD, the Bonferroni-corrected α was 0.167. Based on Cohen’s d, the effect size was 0.6 for Hcy, 0.75 for β2-MG, and 0.5 for CysC. For the subsequent study, the experimental group was divided into four subgroups based on the severity of DR in T2DM patients. The power was set as 0.9, and the initial calculation yielded a total sample size of 96 patients, with 24 patients in each subgroup. However, sensitivity analysis suggested a larger sample size of 140 patients, with 35 patients in each subgroup. Due to the non-normally distributed nature of the data, the Kruskal–Wallis test was utilized. To account for potential variability, the sample size was increased by 10%–20%, resulting in a total of 168 patients. For correlation analysis, Spearman’s rank correlation coefficient (ρs > 0) was employed. Based on 2,000 simulations, with a power of 0.9 and a significance level of α = 0.05, ρ1 was determined to be 0.22 (Hcy), 0.56 (β2-MG), and 0.45 (CysC). The integrated analysis recommended a final total sample size of 201 patients.

### Subjects and ethics

**Figure 1:**
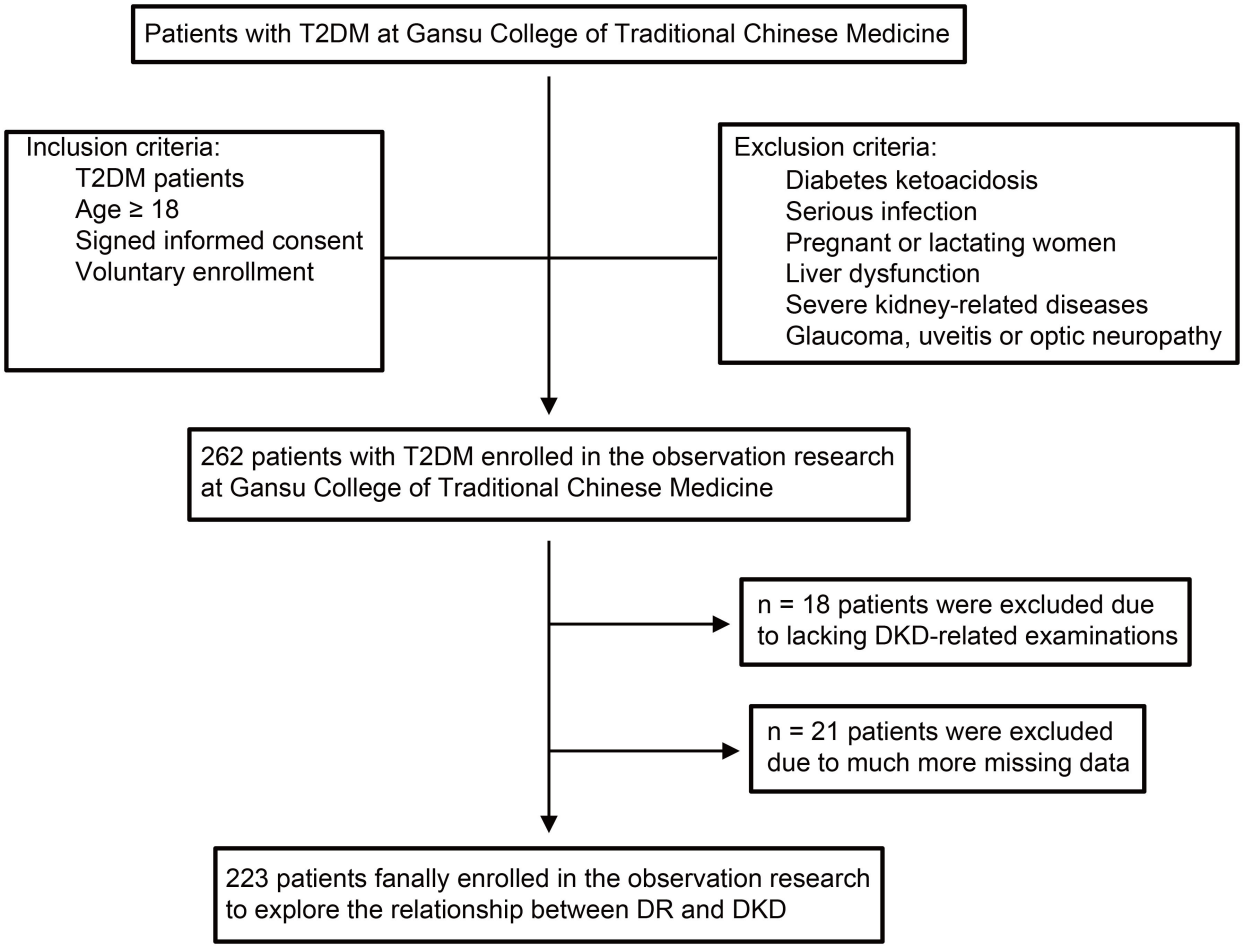
The flow diagram of study participants. The flow diagram shows inclusion and exclusion of patients in the observational research. T2DM, type 2 diabetes mellitus; DR, diabetic retinopathy; DKD, diabetic kidney disease; BP, blood pressure.

Patients with T2DM were recruited from the Gansu Provincial Hospital of Traditional Chinese Medicine between November 2020 and November 2022. Healthy examinees were recruited and used as controls. The diagnostic criteria of T2DM referred to the Guidelines for the Prevention and Treatment of Type 2 diabetes in China (2020 edition) (12). According to a previous study (13), the diagnostic criteria and staging criteria for DR were defined. Three DKD indicators were estimated to determine the occurrence of clinical DKD, namely, CysC, β2-MG, and Hcy. The detailed inclusion and exclusion criteria were available in **Figure 1**. The inclusion criteria specified patients diagnosed with T2DM aged 18 years or older. The exclusion criteria ruled out patients with diabetic ketoacidosis, serious infection, liver dysfunction, severe kidney-related diseases, glaucoma, uveitis, or optic neuropathy, as well as pregnant or lactating women or those participating in other clinical trials. Additionally, after enrollment, T2DM patients were divided into four subgroups based on their DR stage. Importantly, this subgroup classification was not arbitrarily determined but was strictly based on the diagnostic and staging outcomes of DR. TCM syndrome differentiation standards referred to the TCM Diagnosis and Treatment Standards for diabetes Retinopathy issued by the diabetes Branch of the Chinese Society of Traditional Chinese Medicine in 2011 (9). Ethical approval was granted by the ethics committee of the Gansu Provincial Hospital of Traditional Chinese Medicine (Approval Document Number: 2020-130-04). Before enrollment, all patients gave written informed consent to voluntarily participate in this study.

### Laboratory tests

After an 8-hour fasting period, venous blood samples of enrolled patients were collected by professional nurses. Physiological and biochemical indicators were analyzed using an automatic biochemical analyzer (Coulter AU2700; BECKMAN, Los Angeles, California, USA). These included markers of renal injury (namely, CysC, β2-MG, and Hcy), as well as blood sugar, estimated glomerular filtration rate (eGFR), and serum lipids including total cholesterol (TC), triglyceride (TG), high-density lipoprotein cholesterol (HDL-C), and low-density lipoprotein cholesterol (LDL-C). Glycosylated hemoglobin (HbA1c) was measured using a blood analyzer (Sysmex XN-9000, Sysmex, Kobe, Japan). Participants were instructed to avoid vigorous exercise and the consumption of high-protein foods for 48 hours prior to testing. After bladder emptying, a 24-hour urine collection was conducted, with the total urine volume accurately recorded. Several indicators in urine were measured using the Cobas 8000 E701 fully automatic chemiluminescence immunoassay analyzer (Roche, Switzerland), including urinary total protein (UTP), albumin, SCr, and the urine protein–creatinine ratio (UPCR).

### Statistical analyses

This study used SPSS v26.0 (SPSS Inc., Chicago, IL, USA) to perform statistical analyses. The measurement data were expressed as mean ± standard deviation, while the count data were presented as percentages or ratios. The normality of data distribution was estimated using a normality test. If the data agreed with the normality distribution, group differences were evaluated using a t-test. If the data did not follow a normal distribution, group differences were evaluated using a non-parametric test. The rank-sum test was used for the ranked data, and the chi-square test for the count data. Spearman’s correlation analysis was conducted to examine the relationships between each DKD-related indicator and DR severity or TCM syndrome types. In all scenarios, a p-value < 0.05 was considered statistically significant.

## Results

### Basic clinical characteristics of enrolled patients

**Figure 2:**
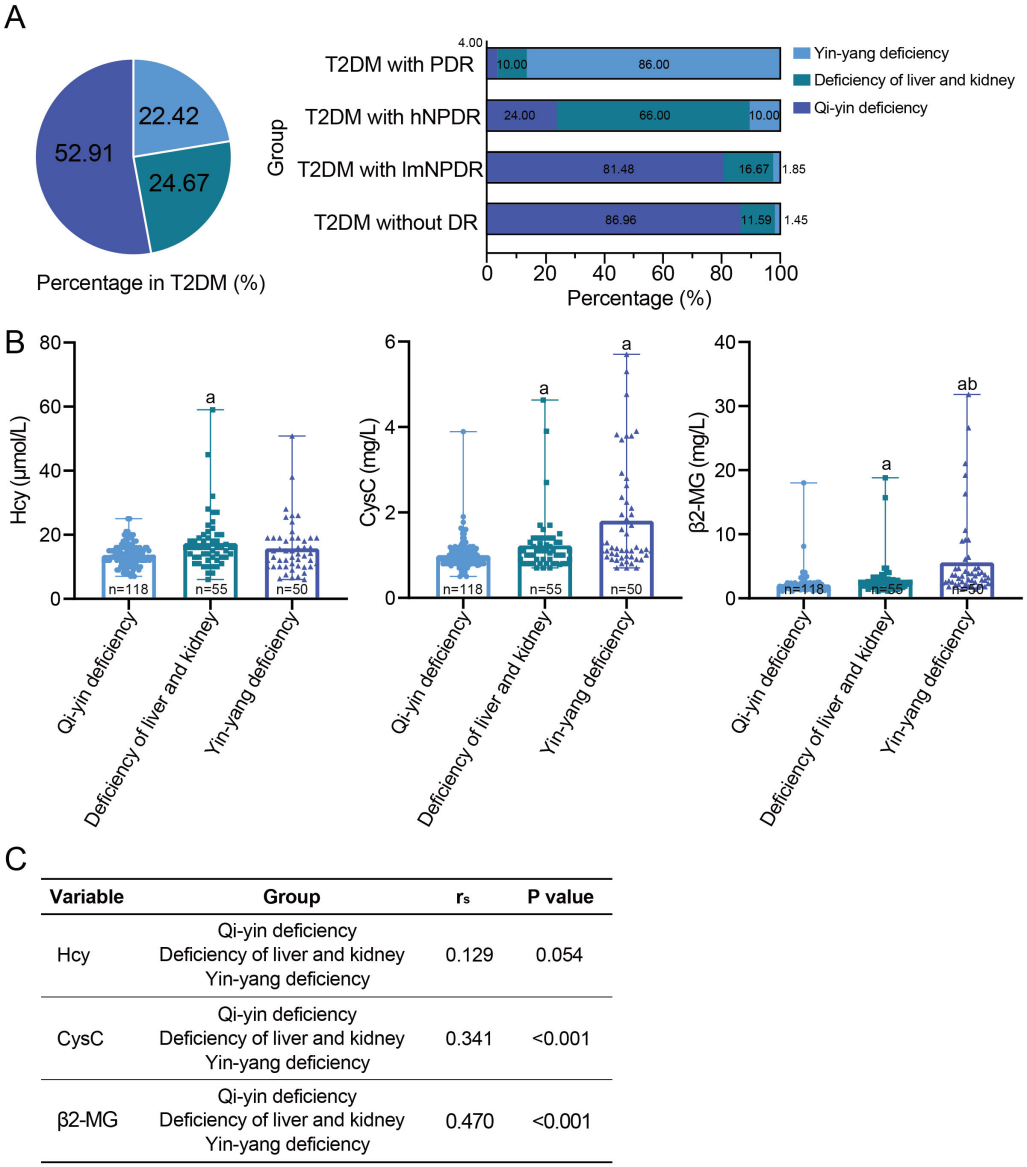
The relationship between the traditional Chinese medicine syndrome types and DR or DKD. **(A)** The traditional Chinese medicine syndrome types in 223 T2DM patients and each T2DM subgroup according to DR staging. **(B)** Comparison of three early DKD indicators among the traditional Chinese medicine syndrome types, namely, Hcy, CysC, and β2-MG. ^a^With statistically significant difference (p < 0.05) compared to the qi–yin deficiency group. ^b^With statistically significant difference (p < 0.05) compared to the deficiency of liver and kidney group. **(C)** Spearman’s correlation between each DKD indicator and the traditional Chinese medicine syndrome types. DR, diabetic retinopathy; DKD, diabetic kidney disease; T2DM, type 2 diabetes mellitus; Hcy, homocysteine; CysC, cystatin C; β2-MG, β2-microglobulin.

This study included 223 T2DM patients, comprising 135 men (60.54%) and 88 women (39.46%), along with 50 healthy controls, including 32 men (64.00%) and 18 women (36.00%). All raw data are available in **Supplementary Tables 1** and **2**. Based on DR staging, the 223 T2DM patients were further categorized into four T2DM subgroups, namely, T2DM without DR, T2DM with light or moderate non-proliferative DR (lmNPDR), T2DM with heavy non-proliferative DR (hNPDR), and T2DM with proliferative DR (PDR), and the distribution of patients was 69 (30.94%), 54 (24.22%), 50 (22.42%), and 50 (22.42%), respectively. No statistically significant differences were observed in age and gender among the groups (p > 0.05) (**Table 1**, **Supplementary Table 3**). Additionally, the age distribution of each T2DM subgroup approximated a normal distribution (**Supplementary Figure 1A**), further supported by normal quantile–quantile plots (**Supplementary Figure 1B**). The normality test confirmed that the age data followed a normal distribution (**Supplementary Figure 1C**, p > 0.05). However, significant differences were observed in the duration of T2DM and body mass index (BMI) between each T2DM subgroup and the control group (**Figure 2**, **Table 1**, and **Supplementary Table 3**). As to several physiological and biochemical indicators, as well as eGFR, no statistically significant difference was discovered between each of those four subgroups and the control group, except for HbA1c (p < 0.001), UPCR (p < 0.001), HDL (p = 0.006), and LDL (p = 0.001) (**Table 1**, **Supplementary Table 3**). Notably, 154 T2DM patients (154/223, 69.06%) were diagnosed with DR, with a proportion close to 70% (**Supplementary Table 1**). In terms of therapeutic choices, a total of seven medication strategies were identified among the enrolled T2DM patients (**Supplementary Figure 2A**, **Supplementary Table 1**). The proportion of each medication strategy varied slightly across the T2DM subgroups (**Supplementary Figure 2B**). Pearson’s chi-squared test indicated no statistically significant differences in medication strategies between the T2DM subgroups (**Supplementary Figure 2C**).

**Table 1:** Subject demographics and clinical characteristics in the prospective observational research study.

Characteristics	Control (n = 50)	T2DM without DR (n = 69)	T2DM with lmNPDR (n = 54)	T2DM with hNPDR (n = 50)	T2DM with PDR (n = 50)	p-Value
Age (years)	57.18 ± 5.21	58.87 ± 6.58	58.44 ± 6.22	58.72 ± 9.56	57.64 ± 11.71	0.779
Male	32	40	31	34	30	0.773
Female	18	29	23	16	20	
Duration of T2DM (years)	0	8.00 (5.00,10.00)	10.00 (8.00,15.25)	10.00 (4.00,15.00)	15.00 (9.50,20.00)	<0.001
BMI (kg/m^2^)	21.81 ± 1.80	23.03 ± 2.89	23.85 ± 3.61	23.55 ± 2.72	23.52 ± 2.87	0.003
SBP (mmHg)	129.90 ± 12.37	131.54 ± 8.18	131.75 ± 10.83	131.88 ± 8.70	132.52 ± 11.16	0.769
DBP (mmHg)	77.50 ± 9.22	80.46 ± 7.05	84.13 ± 8.45	78.16 ± 7.33	80.42 ± 8.31	0.129
HbA1c (%)	4.96 ± 0.56	8.25 ± 1.45	8.50 ± 1.65	7.82 ± 1.61	8.54 ± 2.02	<0.001
UPCR	374.18 ± 112.59	511.78 ± 121.75	522.28 ± 96.50	515.03 ± 85.66	500.34 ± 81.27	<0.001
SCr (mmol/L)	72.45 (60.30,84.85)	78.50 (65.80,90.80)	75.25 (65.07,87.60)	72.40 (63.80,86.92)	86.7 (65.20,104.52)	0.080
eGFR (mL/min/1.73 m^2^)	107.00(97.00,114.25)	92.00 (78.50,117.00)	101.00(85.00,125.25)	94.50 (76.00,115.25)	104.50 (82.50,117.75)	0.092
TC (mmol/L)	4.27 ± 0.65	4.26 ± 0.53	4.22 ± 0.61	4.30 ± 0.56	4.16 ± 0.66	0.802
TG (mmol/L)	1.22 ± 0.30	1.24 ± 0.28	1.24 ± 0.27	1.21 ± 0.27	1.23 ± 0.27	0.982
HDL (mmol/L)	1.44 ± 0.38	1.28 ± 0.23	1.24 ± 0.27	1.33 ± 0.35	1.39 ± 0.33	0.006
LDL (mmol/L)	2.14 ± 0.46	1.82 ± 0.44	1.94 ± 0.41	1.97 ± 0.37	2.04 ± 0.46	0.001

BMI, body mass index; SBP, systolic blood pressure; DBP, diastolic blood pressure; HbA1c, glycosylated hemoglobin; UPCR, urine protein–creatinine ratio; SCr, serum creatinine; eGFR, glomerular filtration rate; TC, total cholesterol; TG, triglyceride; HDL, high-density lipoprotein cholesterol; LDL, low-density lipoprotein cholesterol; lmNPDR, light or moderate non-proliferative DR; hNPDR, heavy non-proliferative DR; PDR, proliferative DR.

### Higher DKD indicators in T2DM patients

**Figure 3:**
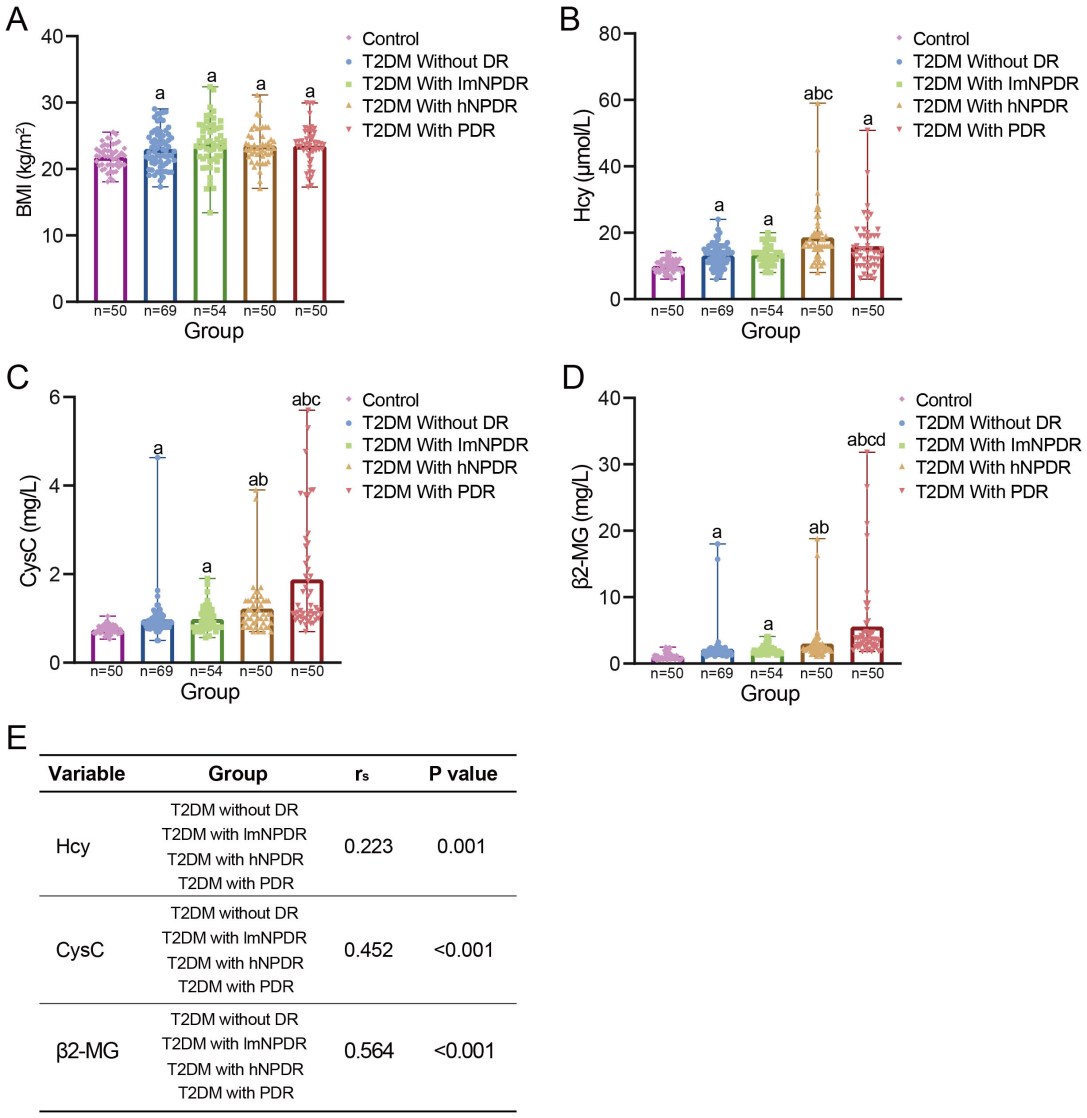
The relationship between DR and DKD. **(A)** Comparison of BMI between control group and each T2DM subgroup. Four T2DM subgroups were divided according to DR staging. ^a^With statistically significant difference (p < 0.05) compared to the control group. **(B–D)** Comparison of three early DKD indicators between control group and each T2DM subgroup, namely, Hcy, CysC, and β2-MG. ^a^With statistically significant difference (p < 0.05) compared to the control group. ^b^With statistically significant difference (p < 0.05) compared to the subgroup of T2DM without DR. ^c^With statistically significant difference (p < 0.05) compared to the subgroup of T2DM with lmNPDR. ^d^With statistically significant difference (p < 0.05) compared to the subgroup of T2DM with hNPDR. **(E)** Spearman’s correlation between each DKD indicator and T2DM subgroups. BMI, body mass index; T2DM, type 2 diabetes mellitus; DR, diabetic retinopathy; DKD, diabetic kidney disease; Hcy, homocysteine; CysC, cystatin C; β2-MG, β2-microglobulin; lmNPDR, light or moderate non-proliferative DR; hNPDR, heavy non-proliferative DR.

All three key DKD indicators, namely, Hcy, CysC, and β2-MG, showed statistically significant differences between each T2DM subgroup and the control group (**Figures 3B**, **Supplementary Table 3**). Furthermore, as the severity of DR increased, the levels of these DKD indicators exhibited a generally upward trend, with statistical significance for all indicators except for the Hcy indicator in the PDR subgroup (**Figures 3B**, **Supplementary Table 3**). Spearman’s correlation analysis revealed significant correlations between each DKD indicator and the T2DM subgroups, with rs = 0.223, p = 0.001, for Hcy; rs = 0.452, p < 0.001, for CysC; and rs = 0.564, p < 0.001, for β2-MG (**Figure 3**). Additionally, when considering DR staging, we also estimated the effect sizes for each DKD indicator, as well as for the duration of T2DM (**Supplementary Table 3**). The results showed that the duration of T2DM had a medium effect size (Cohen’s d = 0.274). Each DKD indicator presented a higher Cohen’s d value, with 0.348 for Hcy, 0.513 for CysC, and 0.718 for β2-MG. Hcy had a medium effect size. Both CysC and β2-MG demonstrated large effect sizes.

### Traditional Chinese medicine syndrome types in T2DM patients

Three traditional Chinese medicine syndrome types were identified among the 223 T2DM patients, including qi–yin deficiency, deficiency of the liver and kidney, and yin–yang deficiency. Qi–yin deficiency accounted for 52.91% (118/223), followed by deficiency of the liver and kidney (24.67%, 55/223) and yin–yang deficiency (22.42%, 50/223) (**Figure 2**). In particular, the proportion of qi–yin deficiency increased as the severity of DR decreased, while the proportion of yin–yang deficiency showed the opposite trend and increased with the increasing severity of DR (**Figure 2**). As to the three DKD indicators, statistically significant differences were observed between deficiency of the liver and kidney or yin–yang deficiency and qi–yin deficiency, except for Hcy in yin–yang deficiency (**Figure 2**, **Supplementary Table 4**). Furthermore, Spearman’s correlation analysis revealed significant correlations between CysC and traditional Chinese medicine syndrome type, as well as between β2-MG and traditional Chinese medicine syndrome type, with rs = 0.341, p < 0.001, for CysC, and rs = 0.470, p < 0.001, for β2-MG (**Figure 2**). However, no statistically significant correlation was found between Hcy and traditional Chinese medicine syndrome type, with rs = 0.129, p = 0.054, for Hcy (**Figure 2**). Additionally, when considering TCM syndrome types, we also calculated the effect sizes for each DKD indicator (**Supplementary Table 4**). The results showed that Cohen’s d value presented some variations among the three DKD indicators, with 0.19 for Hcy, 0.351 for CysC, and 0.538 for β2-MG. Hcy had a small effect size, CysC had a medium effect size, and β2-MG possessed a large effect size.

## Discussion

Diabetes is increasingly recognized as a global public health crisis, and the prevalence of diabetes will affect over 640 million people in 2040, according to the International Diabetes Federation (14). As two prevalent complications of diabetes (15), DR and DKD share similar pathological and physiological changes and pathogenesis (3). Previous studies have found that T2DM patients with DR and T2DM patients with DKD exhibit overlapping TCM syndrome types (16). Moreover, clinical treatments using Chinese herbal medicine have been shown to effectively alleviate DR when tailored to these TCM syndromes (10, 11). Therefore, understanding the clinical relationship between DR and DKD, as well as the role of TCM syndromes, is crucial for developing novel strategies to simultaneously treat DR and DKD in T2DM patients.

In this study, we performed a prospective observational research study and found that three key DKD indicators in the T2DM group, namely, Hcy, CysC, and β2-MG, showed significantly higher levels than those in the control group (**Figure 3**). Furthermore, these indicators exhibited a generally increasing trend with the increasing severity of DR across T2DM subgroups (**Figures 3B**), highlighting a strong correlation between DR and DKD. Earlier studies have noted that patients with DR are 10 times at risk of developing renal dysfunction, where the occurrence of DKD increased exponentially with the severity of DR, with patients with NPDR at 19.2%, as well as up to 41.2% in those with PDR (17). Recent findings also showed that over 45% of DKD patients exhibited various stages of DR and that T2DM patients with both DR and DKD were subjected to increased overall mortality risk (18). When evaluating effect sizes, β2-MG demonstrated the highest Cohen’s d value, indicating a big effect size (**Supplementary Table 3**). However, the current gold standard of DKD diagnosis is renal biopsy. Given the non-invasiveness and effective nature of β2-MG testing, larger multi-center studies are needed to confirm the robustness of β2-MG in clinical practice. Taken together, these findings support that DR and DKD could be potentially predicted and can be used to diagnose each other, especially for the clinical significance of β2-MG. This reinforces the theory of “the same treatment of different diseases” in T2DM patients.

Regarding TCM syndrome types, three distinct types were discovered, namely, qi–yin deficiency, deficiency of the liver and kidney, and yin–yang deficiency. Interestingly, there existed a relationship of “as one falls, another rises” between yin–yang deficiency and qi–yin deficiency, with the severity of DR decreasing (**Figure 2**), highlighting the pivotal role of TCM syndrome types in DR. However, no clear trend was observed for deficiency of the liver and kidney. Recent studies have demonstrated that over 50% (230/416) of patients with T2DM and DR can be divided into three different traditional Chinese medicine syndrome types (19), aligning with the findings of this study. As to DKD, the three key indicators showed an obvious trend among the above-mentioned three types. Patients with qi–yin deficiency possessed lower levels of DKD indicators, while those with yin–yang deficiency had higher levels, except for Hcy (**Figure 2**). Furthermore, a statistically significant difference was discovered by Spearman’s correlation analysis between DKD indicators and traditional Chinese medicine syndrome types, except for Hcy (**Figure 2**). These results underscore the significant influence of TCM syndrome types on DKD. However, no prior research has been reported yet based on a literature review. Notably, when exploring the relationship between DKD and TCM syndrome types, β2-MG still presented the highest Cohen’s d value (**Supplementary Table 4**), supporting its large effect size. This suggests that β2-MG holds substantial potential as a key biomarker for assessing DKD, DR, and TCM syndrome types in T2DM patients after pending validation through larger multi-center studies. Collectively, the findings demonstrate that different TCM syndrome types are closely related to the severity of DR and DKD, fully supporting the same treatment for different diseases based on TCM syndrome types.

The onset of DKD is often insidious. Currently, the diagnosis of DKD primarily relies on indicators such as the urinary albumin-to-creatinine ratio (ACR) or eGFR, with renal biopsy remaining the gold standard (6). However, these diagnostic methods are often time-consuming or invasive. Some scholars have proposed including DR as one of the diagnostic criteria for DKD, but the concomitant relationship between DR and DKD has not yet been conclusively established. Compared to DKD, the diagnosis of DR mainly relies on non-invasive fundus examination (20), and its diagnostic criteria are more clearly defined and widely accepted. Therefore, further exploration of the association between DR and DKD may help optimize the diagnostic process and classification of DKD. This study confirmed a potential relationship between DR and DKD, as well as between TCM syndrome types and DKD. Notably, β2-MG demonstrated a large effect size, with the highest Cohen’s d value compared to that of Hcy and CysC. If validated in larger multi-center studies, these findings could guide clinical practice in several ways. First, β2-MG should be considered as one of the diagnostic criteria of DKD. Importantly, a suitable cut-off value of β2-MG needs to be determined through multi-center studies, as it shows greater potential compared to other DKD-related indicators. Second, the diagnosis and classification of DR, DKD, and TCM syndrome types should be routinely integrated into the management of T2DM patients. This would enable the stratification of T2DM patients into distinct subgroups for more targeted treatment. Finally, the theory of “the same treatment of different diseases” represents a promising research direction for T2DM patients. Currently, we are conducting a study on the use of traditional Chinese medicine to simultaneously address diabetes-related microvascular complications, with the aim of evaluating its efficacy and safety in improving both fundus and renal function indicators in patients with diabetes complications.

A key strength of this study is its meta-analysis for DR and DKD in T2DM patients, allowing us to assess their relationship from the perspective of TCM. This study provides evidence that DR and DKD could potentially be used to diagnose each other and be effectively alleviated based on TCM syndromes. Nevertheless, several limitations should be acknowledged. First, this study only includes clinical indicators at the time of enrollment, and longer-term follow-up data may be necessary to fully understand the progression of T2DM and its complications. That said, the enrolled patients exhibited varying degrees of DR severity, which to some extent reflected the developmental stages of DR. Second, while this study has revealed a correlation between DR and DKD, the efficacy of simultaneously treating DR and DKD in T2DM patients still requires validation through clinical intervention studies. We are currently conducting research to explore the theory of “the same treatment of different diseases” in T2DM patients. Finally, due to the clinical nature of this study, the absence of *in vitro* animal experiments limits our ability to investigate the underlying pathological mechanisms and crosstalk between DR and DKD. Therefore, future research incorporating fundamental scientific experiments is essential to address these questions.

In conclusion, this study provides evidence that DR and DKD could potentially be used to diagnose each other and that the prevalence of qi–yin deficiency and yin–yang deficiency shows opposite trends with respect to DR severity. These findings suggest that DR and DKD may be effectively managed based on TCM syndromes, offering a compelling case for the integrated treatment of these conditions within the framework of TCM.

## Data Availability

The original contributions presented in the study are included in the article/**Supplementary Material**. Further inquiries can be directed to the corresponding author.

## Supplementary material

Supplementary Figure 1 The medication strategies of the enrolled T2DM patients. **(A)**, the medication strategies of each experimental (T2DM) subgroup based on DR stage. Seven medication strategies were found in four T2DM subgroups, namely subgroup of T2DM without DR, subgroup of T2DM with lmNPDR, subgroup of T2DM with hNPDR and subgroup of T2DM with PDR. **(B)**, the proportion of each medication strategy in each T2DM subgroup. **(C)**, Pearson’s chi-squared test of seven medication strategies between T2DM subgroups. The expected counts of 16 cells (57.1%) was less than five. The minimum expected count was 0.22. The P value was 0.637 more than 0.05, demonstrating comparability among groups.

Supplementary Figure 2 The age distributions and their normality test of the enrolled T2DM patients. **(A)**, the age distributions of T2DM patients in each experimental subgroup based on DR stage. **(B)**, normal Quantile-Quantile plot using ages of T2DM patients in each experimental subgroup based on DR stage. **(C)**, normality test of the age data using Kolmogorov-Smirnov test or Shapiro-Wilk test.
