# Supplementary figures and images for: The association of diabetic retinopathy and diabetic kidney disease in patients with type 2 diabetes mellitus: a prospective observational research study
Source: Front Endocrinol (Lausanne). 2025 Sep 1;16:1473517. doi: 10.3389/fendo.2025.1473517 (PMC12433849; doi:10.3389/fendo.2025.1473517)

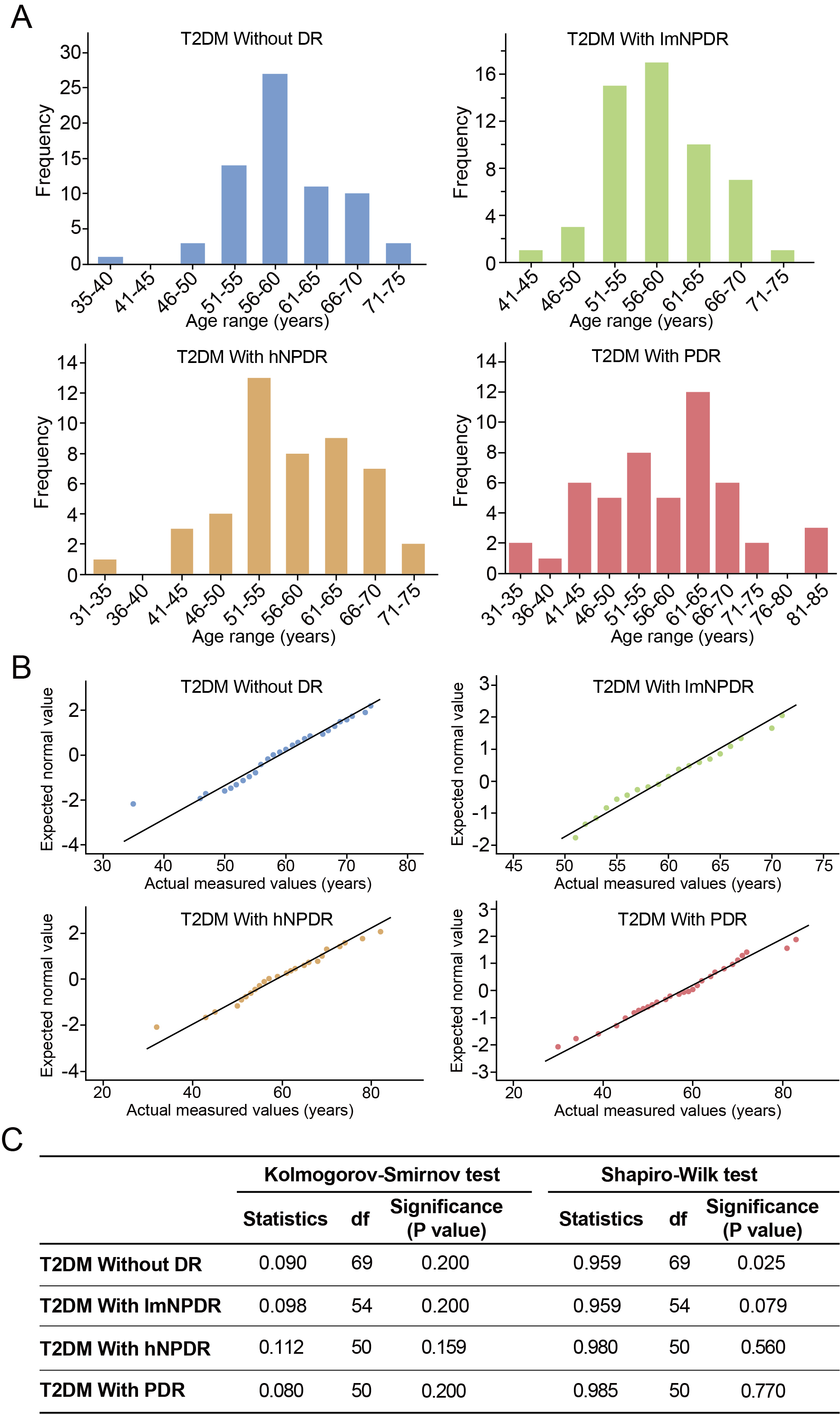

Supplement: Supplementary Figure 1 — The medication strategies of the enrolled T2DM patients. (A), the medication strategies of each experimental (T2DM) subgroup based on DR stage. Seven medication strategies were found in four T2DM subgroups, namely subgroup of T2DM without DR, subgroup of T2DM with lmNPDR, subgroup of T2DM with hNPDR and subgroup of T2DM with PDR. (B), the proportion of each medication strategy in each T2DM subgroup. (C), Pearson’s chi-squared test of seven medication strategies between T2DM subgroups. The expected counts of 16 cells (57.1%) was less than five. The minimum expected count was 0.22. The P value was 0.637 more than 0.05, demonstrating comparability among groups. [file Image1.jpeg]

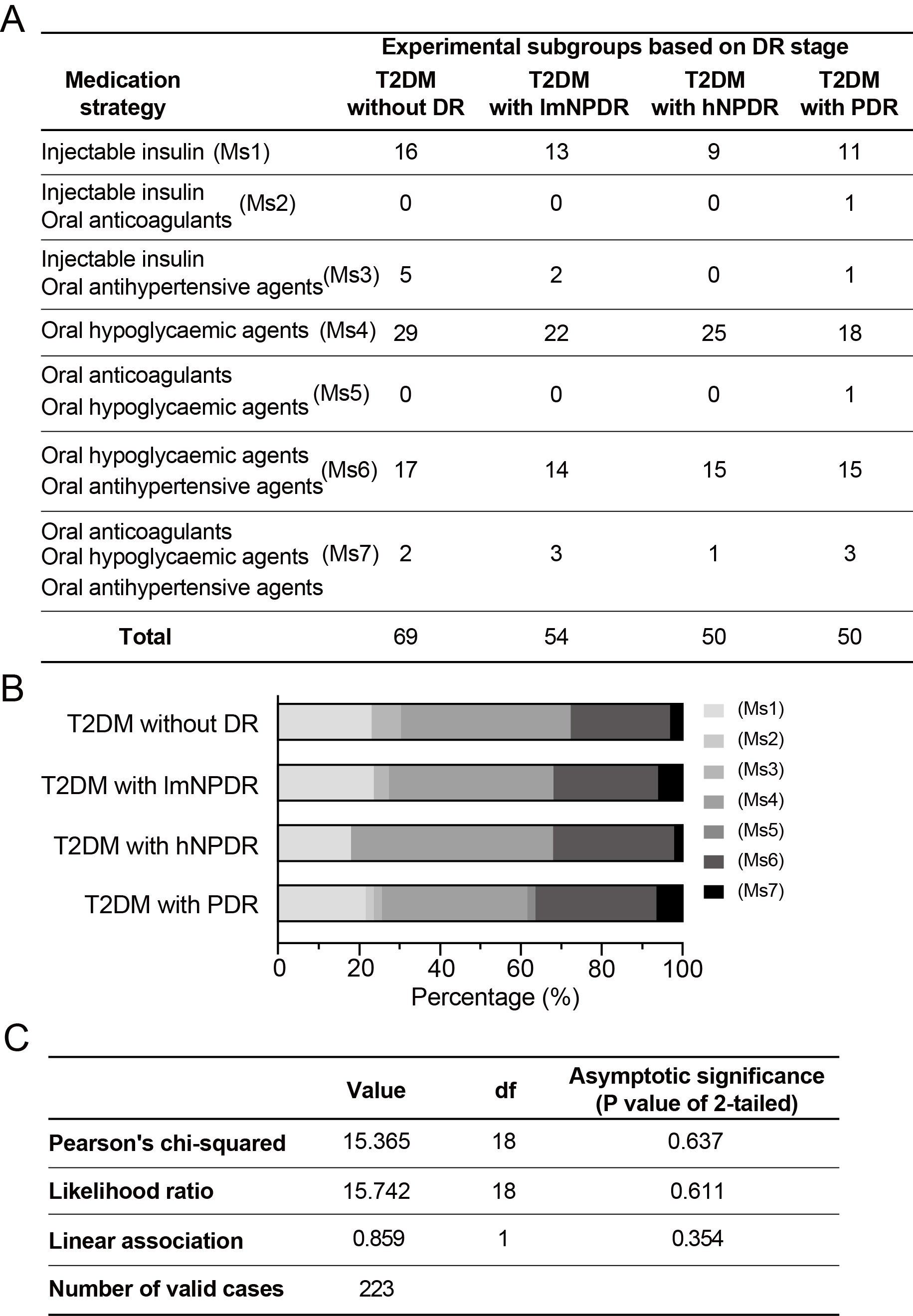

Supplement: Supplementary Figure 2 — The age distributions and their normality test of the enrolled T2DM patients. (A), the age distributions of T2DM patients in each experimental subgroup based on DR stage. (B), normal Quantile-Quantile plot using ages of T2DM patients in each experimental subgroup based on DR stage. (C), normality test of the age data using Kolmogorov-Smirnov test or Shapiro-Wilk test. [file Image2.jpeg]
